# Possible involvement of α1-adrenergic receptor and K_ATP_ channels in cardioprotective effect of remote aortic preconditioning in isolated rat heart

**DOI:** 10.4103/0975-3583.70917

**Published:** 2010

**Authors:** Rajeev Taliyan, Manjeet Singh, Pyare Lal Sharma, Harlokesh Narayan Yadav, Kulwinder Singh Sidhu

**Affiliations:** *Department of Pharmacology., I.S.F College of Pharmacy, Moga, Punjab – 142 001, India*

**Keywords:** Cardio-protection, ischemic preconditioning, ischemia / reperfusion injury, remote aortic preconditioning

## Abstract

**Background:**

Remote preconditioning is a phenomenon in which brief episodes of ischemia and reperfusion to remote organs protect the target organ against sustained ischemia/reperfusion (I/R)-induced injury. Protective effects of remote aortic preconditioning (RAPC) are well established in the heart, but their mechanisms still remain to be elucidated.

**Objective:**

This study has been designed to investigate the possible involvement of α-1-adrenergic receptor (AR) and K_ATP_ channels in cardio-protective effect of RAPC in isolated rat heart.

**Materials and Methods:**

Four episodes of ischemia and reperfusion, each comprising of 5 min occlusion and 5 min reperfusion, were used to produce RAPC. Isolated perfused rat heart was subjected to global ischemia for 30 min followed by reperfusion for 120 min. Coronary effluent was analyzed for LDH and CK-MB release to assess the degree of cardiac injury. Myocardial infarct size was estimated macroscopically using TTC staining.

**Results:**

Phenylephrine (20 μ/kg i.p.), as α-1-AR agonist, was noted to produce RAPC-like cardio-protection. However, administration of glibenclamide concomitantly or prior to phenylephrine abolished cardioprotection. Moreover, prazocin (1 mg/kg. i.p), as α-1-AR antagonist and glibenclamide (1 mg/kg i.p), a K_ATP_ channel blocker, abolished the cardioprotective effect of RAPC.

**Conclusion:**

These data provide the evidence that α-1-AR activation involved in cardioprotective effect of RAPC-mediated trough opening of K_ATP_ channels.

## INTRODUCTION

Coronary artery disease represents a global burden on health care resources, and it is the leading cause of morbidity and mortality in the world by 2020.[1] Repeated short episodes of ischemia and reperfusion have been demonstrated to make myocardium transiently more resistant to deleterious effects of prolonged ischemia and this paradoxical form of myocardial adaptation has been termed as ischemic preconditioning.[2] The occlusion of circumflex artery has produced protection of myocardium supplied by left anterior descending coronary artery and this phenomenon is termed as intracardiac preconditioning.[3] Short occlusion of renal[4] abdominal aorta or mesenteric artery[5] has been documented to prevent myocardium against ischemia and reperfusion-induced injury. This phenomenon has been termed as “remote preconditioning” or intraorgan preconditioning or preconditioning at distant site.[6,7] RAPC is well-documented in various animal models, but the molecular mechanism involved in remote preconditioning is still not well defined.

Previous studies reported that norepinephrine is involved in ischemic preconditioning. Depletion of norepinephrine from sympathetic neurons abolishes ischemic preconditioning, and tyramine-induced release of norepinephrine from sympathetic neurons mimics ischemic preconditioning.[8] Ischemic preconditioning is also mimicked by phenylephrine α-1-adrenergic receptor (AR) agonist and blocked by prazocin α_1_-AR antagonist, suggesting that ischemic preconditioning is mediated by α-1-ARs.[9] However, some investigators have reported that adrenergic stimulation or α_1_-AR agonist methoxamine did not precondition the dog heart.[10] Moreover, α_1_-AR blockade did not abolish ischemic preconditioning in the rat heart.[11,12] Thus, the role of α AR in ischemic preconditioning has been a source of controversy.

The activation of α-1-ARs has been shown to hydrolyse phosphoinositides and produce diacylgleserol (DAG). Hydrolysis of phosphoinositides can lead to mobilization of calcium and production of diacylglycerol, which together are proposed to activate protein kinase C (PKC). The PKC is known to activate K_ATP_ channels and precondition the myocardium.[13,14]

Therefore, this study has been designed to investigate the effect of α-1-AR and K_ATP_ channels in cardioprotective effect of remote aortic preconditioning (RAPC).

## MATERIALS AND METHODS

Wister albino rats of either sex weighing 200–300 were employed in this study. The animal experiments were conducted in accordance with guidelines of US National Institute of Health for care and use of laboratory animals and the study protocol was approved by Institutional Ethics Committee.

### Induction of remote aortic preconditioning

Induction of RAPC was carryout out according to earlier reported by Singh and Sharma.[15] In brief, each rat was anesthetized with thiopental sodium (40 mg/kg, i.p.). A 2-cm long incision was given on the abdomen. Lower portion of abdominal aorta was isolated below the point of origin of renal artery, and a silken suture (numbered 5/0) was used to make a shoelace knot to occlude the abdominal aorta and knot was untied for reperfusion. Four episodes of ischemia and reperfusion, each comprising of 5 min occlusion and 5 min reperfusion, were used to produce RAPC.

### Isolated perfused rat heart

In brief, heart was rapidly excised and immediately mounted on Langendorff’s apparatus. Isolated heart was retrogradely perfused at constant pressure of 80 mmHg with Kreb’s Henseleit (KH), maintained at 37 °C, bubbled with 95% O_2_ and 5% CO_2_. Flow rate was maintained at 7–9 mL/min using Hoffman’s screw. The heart was enclosed in a double wall jacket, the temperature of which was maintained by circulating water heated to 37 °C. Global ischemia was produced for 30 min by blocking the inflow of KH solution. It was followed by reperfusion for 120 min. Coronary effluent was collected immediately 30 min after reperfusion for estimation of lactate dehydrogenase (LDH) and 5 min after reperfusion for estimation of creatine kinase (CK-MB).

### Assessment of myocardial infarct size

Infarct size was measured by macroscopic method using TTC-staining dye, and the infracted area reported as the percentage of total ventricular area.[16] In brief, hearts were removed from the Langendorff’s apparatus and both the auricles and the root of the aorta were excised, and the ventricles were frozen. These were then sliced into uniform sections of 2–3 mm thickness and incubated in 1% triphenyltetrazolium chloride (TTC), at 37 °C in 0.2 M Tris buffer (pH 7.4) for 20 min. TTC was converted to red formazone pigment by reduced nicotinamide adenine dinucleotide (NADH) and dehydrogenase enzyme and, therefore, stained the viable cells deep red, while the infracted cells remained unstained or dull yellow. The ventricular slices were placed between two glass plates and a transparent plastic grid with 100 squares in 1 cm^2^ was placed above it. The average area of each slice was calculated by counting the number of squares on either side and similarly the stained and unstained or dull yellow area was counted. The infracted area was expressed as a percentage of the total ventricular area.

### Estimation of lactate dehydrogenase

Lactate dehydrogenase (LDH) was estimated in samples of coronary effluent collected after stabilization and immediately and 30 min after reperfusion using 2,4-DNPH method as described by King.[17]

### Estimation of creatine kinase

Creatine kinase (CK-MB) was measured in samples of coronary effluent after stabilization and 5 min after reperfusion using modified method of Hughes.[18]

### Experimental protocol

Ten groups, each group comprised of six Wistar albino rats, were employed in this study.

**Group I** (Sham control; *n* = 6): Rats were subjected to surgical procedures to isolate abdominal aorta and to pass ligature beneath it, but aorta was not occluded. Hearts were excised 40 min after isolation of aorta and isolated hearts were perfused continuously on Langendorff’s apparatus for 160 min without subjecting them to global ischemia and reperfusion.

**Group II** (Control group; *n* = 6): Rats were subjected to surgical procedures to isolate abdominal aorta, but aorta was not occluded. Hearts were excised 40 min after the isolation of aorta and isolated hearts were perfused on Langendorff’s apparatus and were subjected to global ischemia for 30 min followed by reperfusion for 120 min.

**Group III** (Remote aortic preconditioning group; *n* = 6): Rats were subjected to surgical procedures to isolate abdominal aorta. Four episodes, each episode comprising 5 min occlusion and 5 min reperfusion were carried out of RAPC. Hearts were excised immediately after the last episode of preconditioning, perfuse on Langendorff’s apparatus and were subjected to global ischemia for 30 min followed by reperfusion for 120 min.

**Group IV** (Prazocin treated control group; *n* = 6): Rats were administered prazocin (1 mg/kg, i.p.), a selective α1- antagonist, 1 h before isolation of abdominal aorta. Rest of protocol was the same as described in group II.

**Group V** (Glibenclamide-treated control group, *n* = 6): Rats were administered glibenclamide (1 mg/kg, i.p.) K_ATP_ channel blocker, 2 h before isolation of abdominal aorta. Rest of protocol was the same as described in group II.

**Group VI** (Phenylephrine-treated control group): Rats were administered phenyephrine (1 mg/kg and 20 μg/ kg, i.p.), a selective α-1 agonist, 1 h before isolation of abdominal aorta. Rest of protocol was the same as described in group II.

**Group VII** (Phenylephrine- and glibenclamide-treated group; *n* = 6): Rats were administered glibenclamide and phenyephrine (1 mg/kg and 20 μg/kg, i.p.), a selective α-1 agonist, 2 h and 30 min before isolation of abdominal aorta, respectively. Rest of protocol was the same as described in group II.

**Group VIII** (Prazocin-treated remote aortic preconditioning group; *n* = 6): Prazocin (1 mg/kg, i.p.) was administered to rats 1 h before isolation of abdominal aorta. Rest of protocol was the same as described in group III.

**Group IX** (Glibenclamide-treated remote aortic preconditioning group; *n* = 6): Rats were administered glibenclamide (1 mg/kg, i.p.) 2 h, before isolation of abdominal aorta. Rest of protocol was same as described in group III.

**Group X** (Glibenclamide- and prazocin-treated remote aortic preconditioning group; *n* = 6): Rats were administered glibenclamide (1 mg/kg, i.p.) and prazocin (1 mg/kg, i.p.) 2 h and 1 h, respectively, before isolation of abdominal aorta. Rest of protocol was the same as described in group III.

### Statistical analysis

Values were expressed as mean ± SD for six animals. Oneway ANOVA followed by Dunnett’s test were employed as *post hoc* tests for multiple comparisons. Value of *P* < 0.05 was considered to be statistically significant.

### Drugs and chemicals

Prazocin (10 mg/mL) was purchased from Smart Pharm Pvt Ltd., India. Glibenclamide (Ind-Swift Ltd., Parmanu, India) were dissolved in PEG 400 (Ranbaxy Fine Chemicals Ltd.) before use. Tris buffer was prepared by adding 50 mL of 0.2 M Tris (CDH Chemicals, Delhi, India) in 32.5 mL of 0.2 HCl and volume was made up to 200mL with distilled water. All other reagents used in the study were of analar grade (Glaxo, Mumbai, India).

## RESULTS

### Effect of remote aortic preconditioning on ischemia and reperfusion-induced myocardial injury

**Figure 1:**
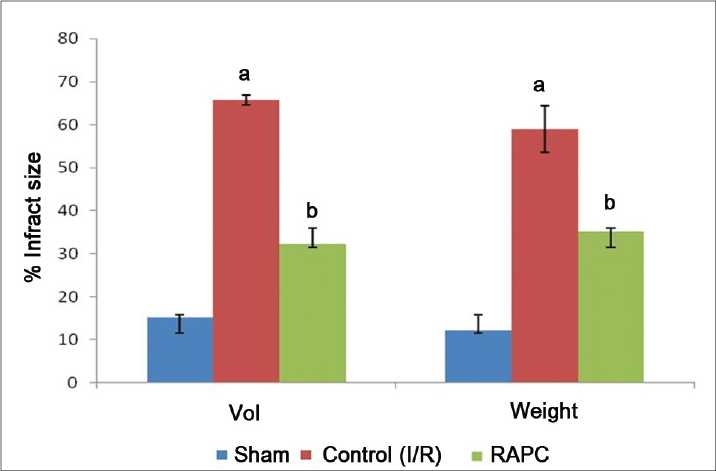
Effect of remote aortic preconditioning (RAPC) on myocardial infarct size measured by volume (vol.) and weight (wt.) method. Results: Each value is expressed as mean ± SD for six animals. *a* = *P* < 0.01 vs. Sham. *b* = *P* < 0.01 vs. control I/R, respectively. I/R = Ischemia and reperfusion, RAPC = remote aortic preconditioning.

**Figure 2:**
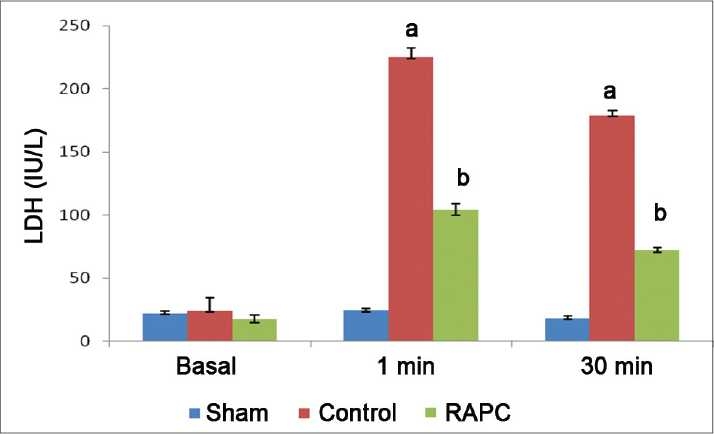
Effect of remote aortic preconditioning (RAPC) on release of lactate dehydrogenase (LDH) in coronary effluent. Results: Values are expressed as mean ± SD for six animals. *a* = *P* < 0.05 vs. Basal. *b* = *P* < 0.05. RAPC: remote aortic preconditioning.

**Figure 3:**
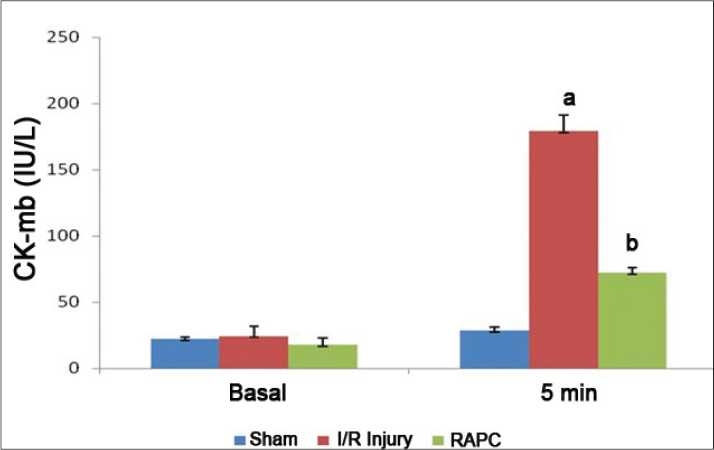
Effect of remote aortic preconditioning on release of creatine kinase (CK-MB) in coronary effluent. Results: Values are expressed as mean ± SD for six animals. *a* = *P* < 0.01 vs. Basal. *b* = *P* < 0.05 vs. I/R injury.

Global ischemia for 30 min followed by reperfusion for 120 min significantly increased myocardial infarct size, release of LDH, and CK-MB in coronary effluent (*P* < 0.05). However, effect of remote aortic preconditioning (RAPC) significantly attenuated ischemia and reperfusion-induced increase in myocardial infarct size (*P* < 0.01), [Figure 1], release of LDH (*P* < 0.05) [Figure 2], and CK-MB (*P* < 0.05) [Figure 3], respectively.

### Effect of pharmacological interventions on cardioprotective effect of remote aortic preconditioning

**Figure 4:**
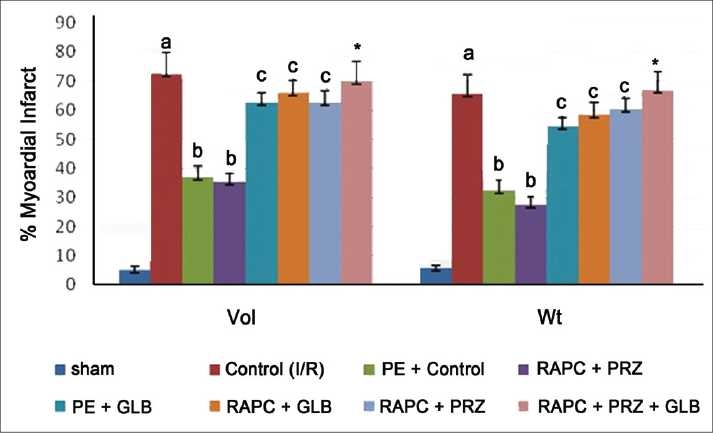
Effect of remote aortic preconditioning (RAPC) and pharmacological interventions on myocardial infarct size. Results: Each value is expressed as mean ± SD for six animals. For volume method: *a* = *P* < 0.05 vs. Sham. *b* = *P* < 0.05 vs. control. c = *P* < 0.05 vs. RAPC, *P < 0.01. For weight methods: *a* = *P* < 0.05 vs. Sham. *b* = *P* < 0.05 vs. control. *c* = *P* < 0.05 vs. RAPC, *P < 0.01. RAPC = remote aortic preconditioning. PE = phenylephrine; Prz = prazocin; Glb = glebenaclamide.

**Figure 5:**
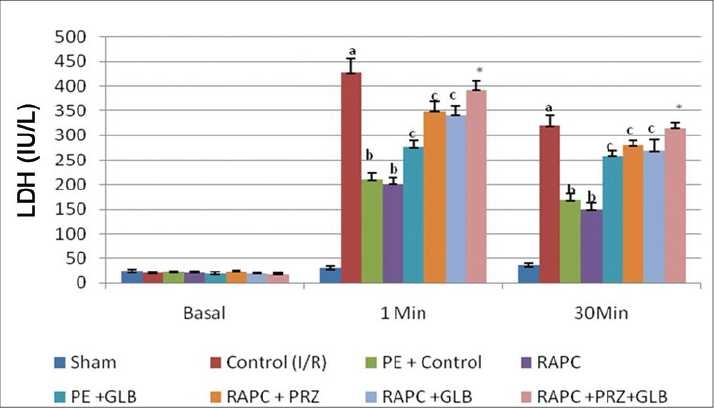
Effect of remote aortic preconditioning (RAPC) and pharmacological interventions on release of lactate dehydrogenase (LDH) in coronary effluent. Values are expressed as mean ± SD for six animals. *a* = *P* < 0.05 vs. Sham. *b* = *P* < 0.05 vs. control (I/R). c = *P* < 0.05 vs. PE and RAPC. *P < 0.01 vs. RAPC. RAPC = remote aortic preconditioning; GLB = glibenclamide; PRZ = prazocin; PE = phenylephrine.

**Figure 6:**
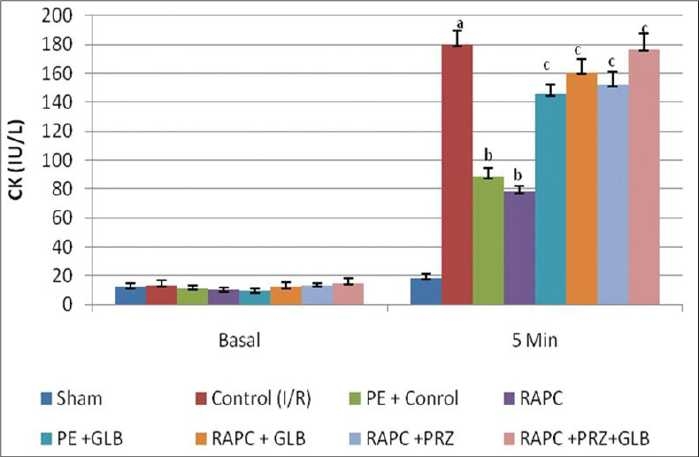
Effect of remote aortic preconditioning (RAPC) and pharmacological interventions on release of creatine kinase (CK-MB) in coronary effluent. Each value is expressed as mean ± SD for six animals. *a* = *P* < 0.05 vs. Sham. *b* = *P* < 0.05 vs. control. c = *P* < 0.05 vs. PE and RAPC. PE = Phenylephrine; PRZ = prazocin; GLB = glibenclamide; RAPC = remote aortic preconditioning.

The administration of prazocin (1 mg/kg, i.p.) and glibenclamide (1 mg/kg, i.p.) produced no marked effect on ischemia and reperfusion-induced increase in myocardial infarct size, release of LDH, and CK-MB in coronary effluent. However, prazocin and glibenclamide significantly prevented RAPC-induced decrease in myocardial infarct size (*P* < 0.01) [Figure 4] release of LDH (*P* < 0.05) [Figure 6] and CK-MB (*P* < 0.05) [Figure 6], respectively. Moreover, administration of combination of prazocin and glibenclamide more markedly prevented RAPC-induced decrease in myocardial infarct size (*P* < 0.01) [Figure 4], release of LDH (*P* < 0.01) [Figure 5], and CK-MB (*P* < 0.01) [Figure 6].

On the other hand, administration of phenylephrine produced RAPC-like cardioprotective effect as compared with control I/R injury rat heart (*P* < 0.05) which was prevented by glibenclamide [Figures 4 and 5]. In addition, phenylephrine also enhanced cardioprotective effect of RAPC (data not shown). Prazocin and glibenclamide given separately and together did not affect coronary flow rate and heart rate (data not shown).

## DISCUSSION

This study demonstrated the involvement of α-1-AR and consequently opening of K_ATP_ channels responsible for cardioprotection afforded by RAPC. The transient occlusion of circumflex artery has been reported to produce protection of myocardial region supplied by left anterior descending coronary artery.[19,20] Moreover, short occlusion and reperfusion of arteries in other anatomical region such as abdominal aorta, limb, and renal occlusions demonstrated to produce cardio-protection against sustained ischemia and reperfusion.[21] Similarly, in this study, four episodes of abdominal aortic preconditioning have significantly attenuated ischemia and reperfusioninduced increase in myocardial infarct size and release of LDH and CK-MB. The peak level of LDH and CK-MB was observed at 0 and 30 min, and CK-MB at 5 min, respectively. This observation is consistent with our previous reports.[22] Langendorff’s preparation and working heart preparation are hemodynamically comparable to investigate the effect of pharmacological agents on ischemia and reperfusion-induced myocardial injury.[23] Moreover, Langendorff’s preparation permits the use of pharmacological interventions without any interference due to changes in systemic circulation.[24] Electrical pacing has not been used in this study because it is reported to release norepinephrine.[25] Therefore, the isolated rat heart preparation, perfuse retrogradely on Langendorff’s apparatus, has been employed in this study.

Activation of a-1-ARs have been reported to produce ischemic preconditioning like cardioprotective effect which is attenuated by selective α-1-adrenergic antagonist-like prazocin.[26] Ischemic episode of short duration are reported to release noradrenaline and produce cardioprotective effect through activation of α-1-AR.[27] Activation of α-1- ARs are noted to activate protein kinase C (PKC) which protect heart from ischemia and reperfusion-induced injury.[28,29] Horeover, prazocin, an α-1-AR inhibitor, has been shown to attenuate the cardioprotective effect of ischemic preconditioning.[30] Therefore, it is possible that α-1-AR activation involved in cardioprotective effect of RAPC. In this study, we observed that prazocin treatment attenuated the cardioprotective effect of RAPC, assessed in terms of myocardial infarct size (Figure 1, *P* < 0.05) and release of LDH (Figure 2, *P* < 0.05) and CK-MB (Figure 3, *P* < 0.05). Therefore, it may be probable to suggest that cardioprotective effect of RAPC may be mediated through activation of α-1-ARs.

Previously, Banerjee *et al*. presented evidence for a role of endogenous norepinephrine in preconditioning rat hearts. They found that administration of norepinephrine or phenylephrine mimicked ischemic preconditioning like cardioprotective effect, whereas reserpine (noradrenaline depletor), phentolamine, and BE-2254 (a selective a-1- adrenoceptor antagonist) block ischemic preconditioninginduced cardioprotection. Moreover, recently it has been noted that α-1b-AR activation alleviates ischemia/ reperfusion (I/R)-induced injury by limiting mitochondrial Ca2+ overload in heart.[31] We found that administration of phenylephrine produced marked cardioprotection as compared with I/R injury control rat heart measured in terms of LDH, CK-MB, and infarct size. However, cardioprotective effect afforded by phenylephrine was abolished in rats received glibenclamide concomitantly or prior to phenylephrine administration. It indicates that a-1-AR activation-mediated preconditioning like cardioprotection may occur through opening of K_ATP_ channels.

Regulation of ion channel through activation of kinases such as protein kinase A (PKA) and PKC is an important mechanism that regulates a wide variety of cellular functions. The phosphorylation by PKA and PKC on serine and threonine residue is known to alter channel properties by modifying the kinetics and/or number of channels present on plasma membrane, including K_ATP_ channels.[32] Classical K_ATP_ consist of inward rectifier Kir6.2 subunits and sulfonylurea receptor subunits (SUR1 or SUR2). The SUR is a member of the ATP-binding cassette (ABC) family of proteins and acts as a regulatory subunit, conferring ADP sensitivity and the distinctive pharmacological characteristics on the K_ATP_ channel complex.[33] On the other hand, the Kir6.x subunit forms the pore of the channel and mediates the defining ATPdependent inhibition of K_ATP_ channels.[34] In addition to being regulated by various nucleotides, K_ATP_ channels are modulated by hormones, noradrenaline, intracellular signals such as G proteins (Gs), phosphatidylinositol-4,5 phosphate (PIP2) that modulate K_ATP_ channel activity.[35] It has been shown that the activities of K_ATP_ channels are regulated also by PKA.[32] Further, in myocardium, the K_ATP_ channels are also activated by Gs-coupled receptor stimulation or by addition of exogenous PKA.[36] Recently, it was observed in animals that the delayed protection following ischemic PC is abolished in vivo by chelerythrine, a PKC inhibitor.[37] PKC is known to modulate K_ATP_ channels.[37] K_ATP_ channels are well reported to be involved in cardioprotection afforded by remote ischemic preconditioning.[38,39] It seems that activation of PKA or PKC modulate K_ATP_ channels that are involved in IP and RAPC.

In this study, we found that glibenclamide, a K_ATP_ blocker, attenuated RAPC-induced cardioprotection (Figures 4 and 5, *P* < 0.05). Our results are fully consistent with previous report by Michael *et al*.[14] In addition, we found that phenylephrine produced cardioprotective effect similar to RAPC, which was abolished by concurrent or prior administration of glibenclamide subjected to preconditioning. Phenylephrine is reported to activate α-1-ARs, and glibenclamide is documented to block K_ATP_ channels.[29,33] Therefore, it seems that the cardioprotective effect of RAPC may be due to activation of α-1-ARs and subsequent opening of K_ATP_ channels. Moreover, it appears that α-1-ARs is working upstream and acts via activation of K_ATP_ channels, which subsequently preconditioning the rat heart.

On the basis of present data, it is concluded that activation of α-1-ARs and consequent opening of K_ATP_ channels may be responsible for the cardioprotective effect of RAPC. Further studies are needed to confirm the exact mechanism involved in RAPC.

## References

[1] Murry CJ, Lopez AD (1997). Alternative projections of mortality and disability by cause 1990-2020: Global burden of disease study. Lancet.

[2] Murry CE, Jennings JB, Reimer KA (1986). Preconditioning with ischemia: A delay of lethal injury in ischemic myocardium. Circulation.

[3] Przyklenk K, Darling CE, Dickson EW, Whittaker P (2003). Cardioprotection ‘outside the box’-the evolving paradigm of remote preconditioning. Basic Res Cardiol.

[4] Pell TJ, Baxter GF, Yellon DM, Drew GM (1998). Renal ischemia preconditions myocardium: Role of adenosine receptors and ATP- sensitive potassium channels. Am J Physiol.

[5] Singh D, Chopra K (2004). Evidence of the role of angiotensin AT1 receptors in remote renal preconditioning of myocardium. Methods Find Exp Clin Pharmacol.

[6] Weinbrenner C, Schulze F, Sarvary L, Strasser RH (2004). Remote preconditioning by infrarenal aortic occlusion is operative via delta1-opioid receptors and free radicals *in vivo* in the rat heart. Cardiovasc Res.

[7] Garrett JG (2005). Remote preconditioning and delayed cardioprotection in skeletal muscle. Am J Physiol Regul Integr Comp Physiol.

[8] Toombs CF, Wiltse AL, Shebuski R (1993). Ischemic preconditioning fails to limit infarct size in reserpinized rabbit myocardium: Implication of norepinephrine release in the preconditioning effect. Circulation.

[9] Tsuchida A, Liu Y, Liu GS, Chosen MV, Downey GM (1994). Alpha-1 adrenergic receptor agonist preconditioning rabbit heart independent of adenosine by direct activation of protein kinase c. Circ Res.

[10] Sebbag L, Katsuragawa M, Verbinski S, Jennings RB, Reimer KA (1996). Intracoronary administration of the alpha 1-receptor agonist, methoxamine, does not reproduce the infarct-limiting effect of ischemic preconditioning in dogs. Cardiovasc Res.

[11] Bugge E, Ytrehus K (1995). Ischemic preconditioning is protein kinase C dependent but not through stimulation of alpha adrenergic or adenosine receptors in the isolated heart. Cardiovasc Res.

[12] Gao XM, Wang BH, Woodcock E, Du XJ (2000). Expression of active α1B-adrenergic receptors in the heart does not alleviate ischemic reperfusion injury. J Mol Cell Cardiol.

[13] Mitchell MB, Meng X, Ao L, Brown JM, Harken AH, Banerjee A (1995). Preconditioning of isolated rat heart is mediated by protein Kinase C. Circ Res.

[14] Michael AM, Patrick DA, Peter CN, Homa A, Ning H, Murtuza Z (2005). Mitochondrial K^ATP^ channels in hindlimb remote ischemic preconditioning of skeletal muscle against infarction. Am J Physiol Heart Circ Physiol.

[15] Singh N, Singh A, Dhalla NS, Angel RA, Pierce GN (2004). Mechanism of cardioprotective effect of remote aortic preconditioning. Pathophysiology of Cradivascular disease.

[16] Fishbein MC, Fishbein S, Rit J, Lando U, Kanmatsuse K, Mercier JC (1981). Early phase acute myocardial infarct size quantification: Validation of triphenyl tetrazolium chloride tissue enzyme staining technique. Am Heart J.

[17] King JA (1959). A routine method for estimation of lactate dehydrogenase activity. J Med Lab Technol.

[18] Hughes BP (1962). A method for the estimation of serum creatine kinase and its use in comparing creatine kinase and aldolase activity in normal and pathological sera. Clin Chim Acta.

[19] Przyklenk K, Baurer B, Ovize M, Kloner RA, Whittaker P (1993). Regional ischemic “preconditioning” protects remote virgin myocardium from subsequent sustained coronary occlusion. Circulation.

[20] Weinbrenner C, Nelles M, Herzog N, Sarvary L, Strasser RH (2002). Remote preconditioning by infrarenal occlusion of the aorta protects the heart from infarction: A newly identified non-neuronal but PKC-dependent pathway. Cardiovasc Res.

[21] Ren C, Gao X, Steinberg GK, Zhao H (2008). Limb remote-preconditioning protects against focal ischemia in rats and contradicts the dogma of therapeutic time windows for preconditioning. Neuroscience.

[22] Sharma A, Singh M (2000). Possible mechanism of cardioprotective effect of ischemic preconditioning in isolated rat heart. Eur J Pharmacol.

[23] Neely JR, Rovetto MJ, Hardman JG, O’Malley BW (1975). Techniques for perfusion isolated rat hearts. Methods in enzymology.

[24] Verdouw PD, van den Deol MA, de Zeeu WS, Dunker DJ (1998). Animal models in the study of myocardial ischemia and ischemic syndromes. Cardiovasc Res.

[25] Flynn SP, Gristwood RW, Owen DA (1978). Characterization of an isolated, working guinea-pig heart including effects of histamine and noradrenaline. J Pharmacol Methods.

[26] Banerjee A, Locke-Winter C, Rogers KB, Mitchell MB, Brew EC, Cairns CB (1993). Preconditioning against myocardial dysfunction after ischemia and reperfusion by an α-adrenergic mechanism. Circ Res.

[27] Fedida D, Braun AP, Giles WR (1993). αl-Adrenoceptors in myocardium: Functional aspects and transmembrane signaling mechanisms. Physiol Rev.

[28] Hu K, Li GR, Nattel S (1995). Mechanisms of ischemic preconditioning in rat: Involvement of α1B-Adrenoceptors, Pertussis Toxin–Sensitive G Proteins, and Protein Kinase C. Circulation.

[29] Roya N, Alireza I, Mahdieh F, Mahdieh F (2010). Phenylephrine produces late pharmacological preconditioning in the isolated rat heart. Eur J Pharmacol.

[30] Rorabaugh BR, Ross SA, Gaivin RJ, Papay RS, McCune DF, Simpson PC (2005). α1A- but not α1B-adrenergic receptors precondition the ischemic heart by staurosporinesensitive, chelerythrine-insensitive mechanism. Cardiovasc Res.

[31] Gao H, Chen OL, Young HT (2007). Activation of (alpha)-1B-adrenoceptor alleviate ischemia/reperfusion injury by limitation of mitochondrial Ca2+ overload in cardiomycytes. Cardiovasc Res.

[32] Pascal B, Kazuaki N, Motoi N, Tohru G, Susumu S (1999). PKA-mediated phosphorylation of the human K_ATP_ channel: Separate roles of Kir6.2 and SUR1 subunit phosphorylation. EMBO J.

[33] Burke MA, Mutharasan RK, Ardehali H (2008). The sulfonylurea receptor, an atypical ATP-binding cassette protein, and its regulation of the K_ATP_ channel. Circ Res.

[34] Tucker SJ, Gribble FM, Zhao C, Trapp S, Ashcroft FM (1997). Truncation of Kir6.2 produces ATP-sensitive K+ channels in the absence of the sulphonylurea receptor. Nature.

[35] Baukrowitz T, Schulte U, Oliver D, Herlitze S, Krauter T, Tucker SJ (1998). PIP2 and PIP as determinants for ATP inhibition of K_ATP_ channels. Science.

[36] Quayle JM, Bonev AD, Brayden JE, Nelson MT (1994). Calcitonin gene-related peptide activated ATP-sensitive K+ currents in rabbit arterial smooth muscle via protein kinase A. J Physiol.

[37] Shi Y, Cui N, Shi W, Jiang C (2008). A short motif in Kir6.1 consisting of four phosphorylation repeats underlies the vascular K_ATP_ channel inhibition by protein kinase C. J Biol Chem.

[38] Shaid M, Tauseef M, Sharma KK, Fahim M (2008). Brief femoral artery ischemia provides protection against myocardial ischemia–reperfusion injury in rats: The possible mechanisms. Exp Physiol.

[39] Stavros PL, Rupert W, Anna TP, Shyamsunder K, Tim JC, Derek MY (2007). Transient limb ischemia induces remote preconditioning and remote postconditioning in humans by a K_ATP_ channel–dependent mechanism. Circulation.

